# Acceptance of Automated Social Risk Scoring in the Emergency Department: Clinician, Staff, and Patient Perspectives

**DOI:** 10.5811/westjem.18577

**Published:** 2024-05-29

**Authors:** Olena Mazurenko, Adam T. Hirsh, Christopher A. Harle, Cassidy McNamee, Joshua R. Vest

**Affiliations:** * Indiana University, Richard M. Fairbanks School of Public Health, Department of Health Policy and Management, Indianapolis, Indiana; † Indiana University, School of Science, Indianapolis, Indiana; ‡ Regenstrief Institute, Center for Biomedical Informatics, Indianapolis, Indiana

## Abstract

**Introduction:**

Healthcare organizations are under increasing pressure from policymakers, payers, and advocates to screen for and address patients’ health-related social needs (HRSN). The emergency department (ED) presents several challenges to HRSN screening, and patients are frequently not screened for HRSNs. Predictive modeling using machine learning and artificial intelligence, approaches may address some pragmatic HRSN screening challenges in the ED. Because predictive modeling represents a substantial change from current approaches, in this study we explored the acceptability of HRSN predictive modeling in the ED.

**Methods:**

Emergency clinicians, ED staff, and patient perspectives on the acceptability and usage of predictive modeling for HRSNs in the ED were obtained through in-depth semi-structured interviews (eight per group, total 24). All participants practiced at or had received care from an urban, Midwest, safety-net hospital system. We analyzed interview transcripts using a modified thematic analysis approach with consensus coding.

**Results:**

Emergency clinicians, ED staff, and patients agreed that HRSN predictive modeling must lead to actionable responses and positive patient outcomes. Opinions about using predictive modeling results to initiate automatic referrals to HRSN services were mixed. Emergency clinicians and staff wanted transparency on data inputs and usage, demanded high performance, and expressed concern for unforeseen consequences. While accepting, patients were concerned that prediction models can miss individuals who required services and might perpetuate biases.

**Conclusion:**

Emergency clinicians, ED staff, and patients expressed mostly positive views about using predictive modeling for HRSNs. Yet, clinicians, staff, and patients listed several contingent factors impacting the acceptance and implementation of HRSN prediction models in the ED.

Population Health Research Capsule What do we already know about this issue?

*The emergency department (ED) has challenges in screening patients for health-related social needs (HRSN). Artificial intelligence based predictive modeling, to determine which patients need social resouces, may address some HRSN screening challenges.*

What was the research question?

*Our goal was to explore the perspective of emergency clinicians, ED staff, and patients on the acceptability and usage of HRSN predictive modeling in the ED.*

What was the major finding of the study?

*Emergency clinicians, ED staff, and patients agreed that artificial intelligence-based predictive modeling, to screen patients for the need for social services, must lead to actions and positive patient outcomes.*

How does this improve population health?

*Prediction models for HRSNs can potentially improve screening and contribute to addressing the HRSN needs of patients in the ED.*

## INTRODUCTION

Screening for, and addressing, patients’ health-related social needs (HRSN) is an increasingly common aspect of patient care^1^^,^^2^ that is supported by numerous professional organizations^3^ and policy makers.^4^^,^^5^ Patients’ HRSNs encompass a variety of nonclinical, socioeconomic, and contextual factors that are essential drivers of morbidity, mortality, utilization, disparities, and costs.^6^^,^^7^ The emergency department (ED) is a potentially appropriate setting for HRSN screening, as a high proportion of ED patients report HRSNs,^8^^–^^11^ patients with HRSN often have difficulty accessing primary care services,^12^ and EDs frequently are the source of care for underserved and vulnerable populations.^13^^,^^14^

The ED presents several challenges to HRSN screening, and patients are frequently not screened for HRSNs.^1^^,^^15^^,^^16^ For example, ED workflows are sometimes unclear about which care team members should screen for or intervene on patients’ HRSNs.^1^^,^^10^^,^^15^^,^^17^ Also, a recent Society for Academic Emergency Medicine panel noted that, given the resources required, it is debatable whether EDs should engage in targeted or universal HRSN screening.^18^ Ideally, HRSN screening should also help identify a course of action for addressing patients’ HRSNs.^19^^–^^21^ Yet clinicians experienced with screening efforts report having insufficient information to refer patients to appropriate services.^22^^,^^23^ As further complication, some patients may decline to share HRSNs they deem stigmatizing or unrelated to their clinical needs.^24^^,^^25^

Predictive modeling using machine learning and artificial intelligence (ML/AI) approaches may address some pragmatic HRSN screening challenges in the ED. Predictive modeling involves applying statistical or computer science methods to healthcare data to prospectively classify patients according to underlying risks.^26^ Predictive models in clinical information systems have demonstrated promise in identifying patients with HRSNs.^27^^–^^29^ Because predictive modeling is automated, it can eliminate some pragmatic challenges, including time constraints, workflow challenges, or staff availability. Also, automated predictive modeling operates as a universal screening program. Thus, it is less susceptible to biases that lead to selectively administered screening questionnaires,^21^ missing data due to patient nonresponse, or omissions in clinical text because clinicians failed to record needs or patients did not disclose them.^30^^–^^32^ Furthermore, predictive modeling can capitalize on the growing volume of data in electronic health records (EHR), health information exchange, and data from non-healthcare organizations that reflect patients’ social circumstances and factors.^33^^,^^34^ This data can provide a longitudinal and comprehensive patient overview and is not dependent on a single healthcare organization for data collection. Finally, the risk scores created by predictive modeling can be the inputs to clinical decision support systems that refer patients to needed services.^29^

Implementing HRSN predictive modeling in ED settings represents a substantial change from current approaches of questionnaire-based screening or collecting HRSN data during patient examinations.^1^ Such changes can elicit mixed reactions from relevant parties, despite their potential advantages. For example, physicians, non-physician clinicians, and healthcare administrators favor explainable predictive models with clear rules; thus, they may be less receptive to advanced prediction models that are less interpretable.^35^ In this study, we explored the acceptability of HRSN predictive modeling by conducting in-depth, semi-structured interviews with emergency clinicians, ED staff, and patients. This study increases understanding of clinician, staff, and patient perceptions of predictive modeling for HSRNs and how predictive modeling could be implemented in ED encounters.

## METHODS

To explore the perceptions of emergency clinicians, ED staff, and patients, we adopted a modified thematic analysis approach^36^ and reported our methods following the Standards for Reporting Qualitative Research (SRQR) recommendations.^37^ The research team had expertise in health informatics, clinical decision support systems, HRSNs, health disparities, and clinical care.

### Context and Sampling Strategy

We recruited emergency clinicians, ED staff, and patients who practiced at or had received care from an urban, Midwest, safety-net teaching hospital system. All research team members have prior or ongoing research collaborations with this healthcare organization. Eligible emergency clinicians included physicians, residents, fellows, and nurse practitioners and were recruited through presentations to faculty groups and emails. Eligible ED staff included social workers, case managers, and registered nurses and were recruited through email in cooperation with organizational leadership. The recruitment presentations and emails provided guidance on how eligible individuals could contact the research team to express their interest in participating in our study. Lastly, we recruited adult (≥18 years old) patients by phone calls to patient representatives identified by the organization’s Community Relations Department and by emails to recent ED patients who had consented to be contacted for research opportunities.

### Data Collection Instruments

Our interview guide included questions to gather perspectives on collecting and using HRSN information through traditional means (eg, survey and discussions with patients). Additionally, the guide asked about the acceptability and usage of predictive modeling for HRSNs in the ED. Because predictive modeling for HRSN would likely be implemented in information technology-based decision support, the interview questions were informed by concepts from two relevant frameworks: the five rights of clinical decision support^38^ framework and the contextual information model.^39^

In our interviews with clinicians and staff, we referenced clinical examples of sepsis risk scoring or opioid use scores. These references were designed to facilitate understanding by drawing parallels to clinical risks often estimated via the application of statistical or computational methods. Like predictive modeling, such scoring approaches leverage multiple patient data elements to arrive at an overall measure of risk. In contrast, we could not assume patients would have the training in, or the direct application of, computational methods to aggregate data to support decisions. Therefore, in our interviews with patients, we referenced online streaming service recommendations or targeted marketing (eg, advertisements or coupons) that draw on prior data collection on consumers to illustrate the application of predictive modeling in everyday experiences.

We piloted the interview guides for length and content with the four members of our study’s advisory panel: a nurse practitioner; a social worker; and two patients. These pilots were not included in the final analytical data. The advisory panel also assists the research team in interpreting the findings in the context of their diverse perspectives and lived experiences. This study is part of a larger project to improve the collection and use of patient health-related social needs in the ED.

### Data Collection Methods

All interviews were conducted using an online meeting platform from December 2022–May 2023. One team member led the interviews of clinicians (physicians and nurse practitioners). A second team member led the interviews of staff (nurses, social workers, and care managers), and the third team member led the interviews of patients. All interviewers were supported by at least one additional team member for notetaking. Interviews lasted, on average, 33 minutes. We met repeatedly during the data collection process to assess the emergence of new information. Saturation was determined when the research team agreed no new themes were being identified. We recorded all interviews with consent for transcription purposes. Before each interview, participants reported age, gender, and race/ethnicity using a web-based survey. Clinicians and staff also reported their credentials and years in practice. We monitored recruitment progress to ensure participant diversity.

### Ethical Issues

All participants provided written consent before data collection. The study was approved by the Indiana University Institutional Review Board.

### Analyses

We analyzed interview transcripts using a modified thematic analysis approach.^36^ Clinician and staff transcripts were analyzed independently from patient transcripts. This decision was based on two considerations: first, clinicians and staff had day-to-day experience with HRSN data collection and applications and, therefore, broader experiences than patients; and second, the results of HRSN screening approaches are predominately clinician-facing; ie, questionnaire results, prediction models, or even interviews during examination are meant to drive decisions and actions of clinicians, not patients. We began with the clinician and staff transcripts. We conducted preliminary screenings of three interview transcripts through a line by line reading process to identify initial themes and confirm that interview questions yielded responses informing our study questions. Once all interviews were completed, we screened all interview transcripts to create an initial codebook. We then tested the codebook reliability by independently applying the codes to three transcripts. We then met and discussed the accuracy and consistency of the codebook and made necessary adjustments. Upon completing the codebook development, three team members consensus coded each transcript. Next, two coders independently coded the same transcripts and then met to adjudicate any differences through discussion to reach consensus.^40^ We agreed on a final set of overarching themes and representative quotes. The above process was repeated on the patient transcripts.

Once all transcripts were consensus coded, we undertook axial coding to identify common, overarching themes. We then met to resolve differences and arrive at a final set of themes. Throughout this process, we employed established procedures in the qualitative methods literature to ensure the rigor and validity of our findings.^41^^–^^43^ These procedures included practicing reflexivity (continually questioning interpretations, seeking answers in the data to verify or challenge interpretations, becoming aware of one’s preconceptions and biases), depth of description (seeking out the rich details of participants’ words), and searching for alternative explanations or interpretations. We used co-occurrence and stratification to compare views about predictive modeling and traditional methods of HRSN information collection. We conducted the entire analysis using Dedoose qualitative analysis software, version 8.2 (SocioCultural Research Consultants, Los Angeles, CA). As a further check on our interpretation, we reviewed a summary of our findings with our advisory panel members.

## RESULTS

Participants included eight emergency clinicians, eight ED staff, and eight patients (Table 1). Participants were mostly female (66.7%) from diverse racial and ethnic backgrounds. The mean age was 42.1 years. Clinician, staff, and patient views of predictive modeling for HRSNs during ED encounters encompassed three broad themes: *impact*; *performance requirements*; and *barriers and facilitators to implementation* (Table 2).

**Table 1.** Demographics of participants.

	Emergency clinicians (n = 8)	ED staff (n = 8)	ED patients (n = 8)	Total (n = 24)
Gender				
Female	50.0	87.5	62.5	66.7
Male	50.0	12.5	25.0	29.2
Transgender	0.0	0.0	12.5	4.2
Race/ethnicity				
Asian	12.5	0.0	0.0	4.2
Black	0.0	37.5	25.0	20.8
Hispanic	0.0	12.5	25.0	12.5
Multiple/other	25.0	12.5	0.0	12.5
White	62.5	37.5	50.0	50.0
Age (mean, SD)	37.8 (7.2)	41.4 (10.9)	47.3 (14.3)	42.1 (11.4)
Work experience (mean years, SD)	7.6 (8.2)	6.1 (5.2)	n/a	n/a

*ED*, emergency department.

**Table 2.** Themes and illustrative quotes from clinicians, staff, and patients on the potential use of risk prediction approaches to health-related social needs in the emergency department setting.

Theme	Description & representative quotes
*Impact*	*Predictive modeling for HRSNs leads to actionable responses to create positive patient outcomes*
Emergency clinician	I think what will solidify it for me is starting to see some positive impact of using that. (#4)
	Knowing the services that are being provided because of this decision. We’re going to increase the number of homeless people off the street and get them into shelters. We’re going to provide this number of patients with food or if we see the value added of that tool, it will get used. If it’s ‘let’s use this tool for the sake of using the tool,’ but we actually don’t see improvement or it actually addresses the unmet need then there will be some hesitation. (#6)
ED staff	Having the algorithm that flags our social work would be more beneficial. Because they could take the time with the patient to set up the resources. Whereas kind of on the medical end, a nurse’s time is thin already. (#15)
Patient	In an ideal world they would connect you with a social worker who would be able to assist you with those things with resources. (#18)
	Stuff that we’ve identified is that this, this, this, and this and we just wanna reach out and see if there’s anything we can do to help you, connect you with resources…It’s gonna get addressed. (#19)
	But I also think that that it could really aid in helping. [Clinicians] see a lot of people, and they have to make a lot of guesses and a lot of judgments on what somebody might need. If it’s my doctor who I’ve been seeing for years, then their guesses are going to be a lot better than somebody seeing somebody in the emergency room for the first time, who has absolutely no record. But, you know, ultimately having some more statistical information to be able to sort through the noise… (#21)
*Performance requirements*	*Details about the functioning of predictive modeling for HRSNs required for acceptance*
Emergency clinician	How up to date is it? How representative of our population is it? How does it keep updating itself over time? If it does all of that very well, then in real-time, it would be updating itself with date, new data every day, and relearning and then reprocessing and then showing up on the EHR. (#1)
ED staff	I would want to know who’s gathering the information. What determines a score? (#13)
	I would probably guarantee that over 50% of patients we see is going to ping this algorithm. (#15)
Patient	I would hope that [risk prediction] wouldn’t discriminate against anyone based on their financial status or anything like that. (#18)
	I think I have the right to know that you’re doing that, you know? I don’t think that you should do it in some secretive fashion and then come to me with these questions when it would be so much easier if you just told me, “Look, you know, we identify certain patterns and -- However they say it, at least let the person know. (#19)
	I just don’t want the computer system just assuming, ‘Oh. She said that she needs public transportation. Oh, that must mean that she has a housing issue’– It doesn’t mean any of that. It’s just, it is what it is. Don’t make apples out of oranges or vice-versa. Just leave it where it is. (#24)
*Barriers and facilitators of implementation*	*Contexts and conditions that would improve adoption and usage*
Emergency clinician	Honestly, being in a teaching hospital, getting the residents onboard first sometimes is easier, 'cause you can get a little bit of upward teaching. If the residents start using it, it kind of forces our attendings to start using it, too. (#2)
ED staff	There’s a lot of creatures of habit that don’t like change. (#12)
Patient	If you have a nurse or a doctor or the medical team or a program or a tablet or anything, …it will be approached in a trusting environment. Because the whole purpose is to help the social need. We really need to make sure is that the approach is friendly and that whoever does it is trained to truly get to the social need, not just to fill out the form, but to make sure and invite the patient, ‘Hey, we want to understand you in our community and we want to help you in every need that you have.’ (#20)

*HRSN*, health-related social needs; *ED*, emergency department; *EHR*, electronic health record.

### Impact

Emergency clinicians, staff, and patients agreed that HRSN predictive modeling should be designed to enable actionable responses and to result in positive patient outcomes. Furthermore, clinician and staff acceptance of predictive modeling tools was contingent on the expectation that routine use of these tools would lead to tangible improvements in patient outcomes. For clinicians and staff, the preference was that predictive modeling would lead to referrals, prompts to collect additional information, and the initiation of connections to services that would change the patient’s health status. As one staff member pointed out:

*“I think it would help … if a score was like generated and…if we had like a dropdown box that had resources…That we can either educate the patient on or give directly to the patient, or coworkers in the hospital like social work, or financial advice that we can send the patient to before they leave the [ED], to kind of get them … on the right track. I feel like we know patients have these issues, but we don’t know how to go about it and … help them.” (#10)*

While clinicians and staff preferred the predictive modeling to support actions, they had mixed opinions about the predictive modeling results being used to initiate automatic referrals to HRSN services. Some participants preferred automatic orders. For example, a physician stated:

“*Whatever you can automate would be ideal.* [EHR] *automatically generates a discharge packet that prints the food voucher and that prints all of the discharge paperwork and then the patient gets it, and they get the referral to primary care, they get the referral to social work, and then it all kind of works out” … (#8)*

Others preferred receiving recommendations they could discard after consulting with the patient, such as described by one nurse:

“*I think having automatic referrals and appointment scheduled would be great, but I also think that it takes a conscious and mindful person when they’re speaking to the patient about everything to go back in and cancel the appointments or change them based off of the patient’s schedule, because some of them they might, might feel offended that, ‘Oh, you’re already making a plan for me. I can take care of myself. I’m grown.’” (#9)*

One physician was strongly against it due to the unknown legal risks: “*When the machine messes up, who, who are we gonna sue? The hospital? The person who coded? The clinician? All of 'em? We don’t have rules for that, yet.”* (#1)

Relatedly, some patients hesitated about automatic referrals to address their HRSNs; rather, they preferred to be consulted on their post-ED care options. This is how one patient described it: *“I don’t want somebody just to automatically take action on it. I want them to just say ‘Here’s what we can offer you.’ Some people feel better about having a shuttle versus taking public transportation…because depending upon the day is depending upon which kind of help I would want.” (#24)*

Additionally, patients reported that results from HRSN prediction models would have the additional benefit of helping initiate conversations about their needs or that assistance would not solely depend upon patients having to disclose sensitive information. This is how a patient described the potential benefits of prediction models:

“*If a person could come due to this algorithm and bring up things that I might not have brought up myself or were reluctant to bring up. Maybe I don’t want to tell people I’m poor. Maybe I don’t want to tell people that we’re struggling at home. Maybe I don’t want to tell people that I just lost my car, because I couldn’t make the payment so I have transportation issues. You know, whatever it is. Everybody’s embarrassment level is different, but yeah, if a nurse could come in and say, you know, “Hey, let me talk to you about this. We have this program. I don’t know if it pertains to you or not, but we have this program and if you are interested, I could probably do something and maybe see if we can get you into it.” (#17)*

### Performance Requirements

Emergency clinicians and staff wanted additional detailed information about HRSN predictive modeling to determine the potential for accepting it in their clinical practice. This additional information included transparency, performance, and concerns for unforeseen consequences. Regarding transparency, emergency clinicians and staff wanted to know the data’s nature, timing, and quality underlying a prediction model. They also wanted to know how often prediction models would be updated based on changes in a patient’s life. As one ED nurse practitioner described it:

“*Is it going to change with new information? Where’s that new information coming from? Six months ago someone may not have had a job and no car, or were living in* [shelter]*, and then now they have a job, they have a subsidized living apartment, they know how to utilize public transport to get around, things like that. Our population is somewhat transient, but you have changes that happen to people that come pretty regularly. And sometimes, it’s positive changes.” (#3)*

A nurse had a similar opinion: “*I would need to know where we got the information from…is it something they filled out on their own?” (#12)*

Like clinicians and staff, ED patients also wanted transparency in how an HRSN prediction model would operate and be used in their care. As one patient put it: “*It would be okay that they’re pulling the information, but I would want to know what that computer system is doing with that information. Are they selling my information? Is it kept in privacy? That would be a big concern.” (#18)*

Clinicians and staff underscored a need for a high-performing prediction model. However, they acknowledged the complexity of HRSN data, as one physician pointed out that “*with anything social, there can be a lot of a gray area.”* (#3) Thus, several clinicians and staff judged prediction model performance in terms of face validity instead of specific performance metrics. This is how one emergency physician explained it:

“*I see something like a risk score* [ie, the product of predictive modeling] *here as a trigger for me to start asking some questions. So, if I go into the room, and I ask a patient about some things, and I’m getting a very confirmatory response there, I think that would probably make me lean more onto a model like that.” (#4)*

Similar to that idea of a “*confirmatory response,*” one physician would check to see whether predictive modeling results “*matches your gestalt.”* (#6) Likewise, a nurse said that she wanted to see that the prediction model “*kind of tracks”* with what she could observe. (#10)

Patients vs clinicians and staff had different perspectives on the negative consequences of poor-performing HRSN predictive modeling. Patients were concerned that prediction models might miss individuals who required services. This is how one patient described it: “*Because that computerized program could pick people up that don’t need to be picked up that really need to be and dismissing people that really need it out.” (#23)*

Furthermore, some patients expressed reservations about potential biases inherent in, or resulting from, predictive modeling. For example, one patient noted the threats if predictive modeling did not account for potential differences in patient background demographics, “*because in that case it doesn’t help. It just becomes an extension of an already biased system*.” (#22) Other patients noted that results from the prediction models should not be used to make other assumptions about patients’ needs or to treat patients differently. In contrast, emergency clinicians and staff expressed concerns with potential over-identification and the wasting of resources. One physician stated: “*If I started seeing a trend of my social worker is coming to me, frustrated, because ‘Hey, I’ve doubled my volume of consults, and I’m seeing all these patients, and I can’t do anything for any of them.’ That would be more concerning.” (#4)*

### Barriers and Facilitators to Predictive Modeling Implementation

Participants acknowledged that predictive modeling is a potentially useful method for measuring and acting upon HRSNs. Given their familiarity with clinical risk scores, the emergency clinicians and staff were generally favorable toward the predictive modeling concept. Nevertheless, they did identify several factors and requirements that would facilitate the adoption of HRSN predictive modeling. For example, emergency clinicians noted the value of clinical champions and specific training. A physician noted: “[The] *majority of people who work in our department have a desire to work with underserved populations, and then those people might be open to trying something. Probably having like, a position champion in the department is a good idea.” (#5)* In addition, ED staff indicated that visible positive impact on their patients can facilitate adoption, but that competing demands for time and attention, as well as general inertia, could inhibit it. A nurse described it thusly:

“*Because people get caught up in their everyday life and no one wants to stop what they’re doing to have to learn something else because it feels like, ‘I don’t have time to do that and that’s just gonna slow me down.’” (#16)*

Several patients described the need for health professionals to be trained to be better communicators when asking about HRSNs, in general, or in response to a prediction model being used. This view may have been rooted in prior experiences of feeling like “*just a number*” (#23) to the healthcare system.

## DISCUSSION

Emergency clinicians, ED staff, and patients were mostly positive about using predictive modeling for HRSNs. Their view that predictive modeling is compatible with the healthcare environment was based on their past experiences delivering (other clinical scores) and receiving (consumer experiences) care. Nevertheless, clinicians, staff, and patients raised several key issues that dampened their acceptance of HRSN prediction models in the ED.

First, participants noted that predictive modeling can support increased awareness of HRSNs. But this alone is insufficient to address HRSNs. For maximum impact, it must be complemented by a straightforward course of action for patient care. For example, predictive modeling connected with a decision support system or referral system could help clinicians direct patients to relevant resources more efficiently and effectively.^44^ This theme from the current interviews aligns with prior work in which clinicians emphasized the need for HRSN screening efforts to directly inform clinical decisions, referral pathways, and interventions.^22^^,^^23^ Also consistent with prior literature on HRSN screening,^45^ we found that patients expect beneficial actions resulting from healthcare organizations’ using HRSN risk predictive modeling. Notably, our participants suggested predictive modeling could be an avenue to initiate further HRSN data collection or investigation and serve as a conversation starter, leading to more comprehensive clinical encounters.

Second, participants envisioned predictive modeling as a complement to, rather than a replacement of, the human-to-human component of HRSNs screening efforts. Emergency clinicians and staff wanted to check prediction model recommendations for consistency with clinical expertise, with the option to override automated orders triggered by a patient HSRN when necessary. Similarly, patients stressed that outcomes or recommendations from any prediction models needed to respect and prioritize their autonomy, specifically their preference to decline or tailor services. Ample evidence suggests that even if patients have identified HRSNs, large percentages may not want any services or actions taken on their behalf.^46^ We note that this theme is somewhat in tension with the preceding theme. That is, while pairing predictive modeling with automated referrals or default orders would have efficiencies of scope and scale, it runs the risk of not respecting patient preferences.

It is possible to ease these tensions through processes that ensure human input. For example, predictive modeling could trigger automated messages to patient portals asking about the desirability of services or prompt inquiries from case managers or patient navigators. Such processes would respect patient preferences and clinical expert knowledge and could enhance the safety and acceptability of predictive models.^47^^,^^48^ Still, while incorporating human input could have benefits, it could also introduce other implicit (or explicit) biases into addressing HRSN. Additionally, incorporating clinical expertise into the process increases the workflow redesign and integration burden. Thus, future implementers of HSRN predictive modeling should carefully evaluate both the model outputs and the human use of these outputs for their roles in introducing or mitigating biases.

Relatedly, participants wanted transparency in prediction models. The artificial intelligence and machine learning (AI/ML) communities have made substantial methodological advances in fostering model explainability, often to illustrate the importance of different model inputs or performance under differing circumstances.^49^ While valuable, this is not the type of transparency the healthcare professionals described to foster acceptance and trust. Participants in this study applied expert judgments to both the data sources and the predictions’ perceived reliability. Such expert judgments on inputs and results are a key component to trusting a prediction model in the clinical medicine field.^48^

Whether expert opinion about data inputs and confirmation with clinical experience is just as applicable to HRSNs is not as clear. The HRSNs are not a primary focus in physician and nurse training, which likely contributes to the fact that HRSNs are seldomly and inconsistently documented.^50^ Another potential contributor is that individuals have implicit biases that may cause them to overlook or overemphasize certain patient characteristics.^51^ Thus, trust in the underlying data should not be dismissed. Still, for implementers of more advanced analytic interventions for HRSNs, eventual end-user acceptance may be more realized through actual performance and changes in patient outcomes.

## LIMITATIONS

First, the study responses and discussions may be influenced by the characteristics of participants who agreed to be interviewed for this study. Second, emergency clinicians and staff were all part of a single healthcare system. Thus, our findings may only generalize to similar settings. Third, we used common examples to make predictive modeling salient to our participants. These examples were identified by our advisory panel members during the piloting of the interview guide. Nevertheless, use of different examples could affect perceptions and responses. Relatedly, the AI field is undergoing rapid evolution. As a result, perspectives on ML and other AI-based tools may swiftly transform as individuals accumulate experience with these technologies and engage in ongoing dialogue about them.

## CONCLUSION

Emergency clinicians, ED staff, and patients expressed mostly positive views about using predictive modeling for health-related socal needs. Nevertheless, clinicians, staff, and patients noted several contingent factors impacting the acceptance and implementation of HRSN prediction models in the ED.
